# Anisotropic Compressive Behavior of Metallic Foams under Extreme Temperature Conditions

**DOI:** 10.3390/ma13102329

**Published:** 2020-05-19

**Authors:** Omid Khezrzadeh, Omid Mirzaee, Esmaeil Emadoddin, Emanoil Linul

**Affiliations:** 1 Faculty of Materials and Metallurgical Engineering, Semnan University, Semnan 35131-19111, Iran; omidkhezrzadeh1993@yahoo.com (O.K.); emadoddin@semnan.ac.ir (E.E.); 2 Department of Mechanics and Strength of Materials, Politehnica University of Timisoara, 1 Mihai Viteazu Avenue, 300 222 Timisoara, Romania

**Keywords:** aluminum metallic foams, compressive behavior, cryogenic and high temperature, structure and property anisotropy, strength properties, energy absorption performances

## Abstract

Metallic foams find their applicability in complex systems that operate under both real-life conditions (Earth living conditions) and extreme temperature conditions (low or high temperatures). In this paper, the main mechanical properties of closed-cell aluminum alloy (A356) foams under quasi-static compression loading conditions were determined. In order to investigate the compressive behavior, three orthogonal directions (X, Y, and Z) and three testing temperatures (−196, 25 and 250 °C) were considered. It has been observed that the temperature significantly influences the strength properties and energy absorption performances of the aluminum metallic foams AMFs. Moreover, it was found that microstructural characteristics, such as intrinsic defects (intracellular cavities, micro-pores and thin cell-walls) and structural anisotropy (shape, size and orientation of cells), play a decisive role in the mechanical behavior of AMFs. Moreover, the paper compares the relative percentage change (relative percentage increase and decrease) of the main normalized compressive properties (yield stress, plateau stress, densification stress and the energy absorption) of AMF samples, according to testing temperature and loading direction.

## 1. Introduction

In recent years, metallic foams (MF)s have been used in many engineering fields as lightweight and protective structures due to their high strength properties and outstanding energy absorption capabilities [1,2,3]. Its widespread use in various industries encourages researchers and experts to study the mechanical aspects of these materials [4,5,6]. Due to the advantageous collapse mechanisms of the cell structure, in recent years, aluminum metallic foams AMFs have received more attention than fully dense metal materials (e.g., steel, aluminum, etc.). AMFs are lightweight materials with low-density as well as new enhanced physical, mechanical, thermal and acoustic properties [7,8,9].

AMFs, similar to most of polymeric foams, are anisotropic, which can be manufactured in different ways to obtain structure (geometry) anisotropy or material (mechanical property) anisotropy [10,11,12]. In some research, however, cell geometries, such as cell size and cell morphology, affect the mechanical behavior of porous/cellular materials [13,14,15]. It was found that the main mechanical properties of cellular materials significantly vary with the number of cells or the size of the constituents [16,17,18,19,20,21,22,23]. Ramamurty and Paul reported that the variability in strength properties is related to variance in the foam cell-size by considering the micro-mechanism of deformation in MFs [16]. The statistical results of Zheng et al. [17] show that the relative energy absorption performances of cellular materials can be improved by increasing their cell irregularity, mainly at the transition impact velocity. Manonukul et al. [18] studied the geometry anisotropy and mechanical property isotropy in two directions of cylindrical MF samples with different cell sizes. Their experimental results show that the compression response for both directions are isotropy. The investigation of Sulong et al. [19] revealed weak anisotropy of the perlite-metallic syntactic foam (MSF) mechanical properties. The effective material properties were slightly higher if the material was loaded parallel to the casting direction. The effect of the initial location of the precursor material in the mold, on the foam structure and compression behavior, was investigated by Nosko et al. [20]. The compression tests revealed that structural anisotropy significantly affects the stress–strain behavior and results in dispersion of the collapse stress. Static and dynamic computational simulations have been performed by Vesenjak et al. [21], in order to investigate the material anisotropy effect of MFs. Based on computed tomography data, they found that due to the manufacturing procedure, foam material exhibits orthotropic anisotropy. The cell-shape anisotropy ratio of Al-Si foams, with various relative densities, was studied by Mu et al. [22]. Their results show that MFs loaded in the transverse direction exhibit a lower stress drop ratio. Moreover, MF foams exhibit higher stress and energy absorption values in the longitudinal direction than in the transverse one. Park and Nutt [23] observed that due to the ellipsoidal cell shapes phenomenon within the foam, yield strength was three times higher in the transverse direction than in the longitudinal one.

Regardless of the matrix material (aluminum, zinc, magnesium, steel, titanium, etc.), different teams of researchers have shown that foam anisotropy is considered an important parameter significantly affecting the compressive properties of MFs and MSFs at room temperature [16,17,18,19,20,21,22,23]. As can be seen in the studies mentioned above, the effect of anisotropy were extensively studied, but only under room temperature loading conditions. Also, the direct effect of temperature on the properties of MFs is an interesting field that has been studied. Different researchers investigated the mechanical behavior of the MFs and MSFs under low [24,25,26] or high [27,28,29] testing temperatures, but without offering discussions and results about the influence of the anisotropy of the MFs on their properties. Their experimental results show that the increase in testing temperature directly affects the energy absorption and strength properties of the MF samples. It was also observed that, according to the metal matrix material, the fracture mechanism of the MF structure changed from brittle to ductile at certain specific temperatures [30,31].

To the best knowledge of the authors, there are no reported results and discussions on the effect of anisotropy on the mechanical behavior of AMFs under extreme operating temperatures. Therefore, this study aims to investigate the quasi-static compressive behavior and compare the mechanical properties and energy absorption performances of AMF samples under different loading conditions and different testing temperatures (cryogenic, room and high temperatures). For this purpose, three orthogonal directions (X, Y, and Z) were taken into consideration, while −196, 25 and 250 °C were the chosen testing temperatures. In addition, according to testing temperature and loading direction, the relative percentage increase and decrease of the normalized properties were presented.

## 2. Materials and Methods

### 2.1. Materials and Sample Preparation

The closed-cell aluminum metal foams (AMFs) were cast in a liquid state process using the foaming method. To this end, the aluminum alloy A356 was melted in a stainless steel mold at 700 °C, while, in order to increase its viscosity, 2 wt.% calcium granule was added to the melted aluminum. The melt was stirred for 10 min at 500 rpm and then 1 wt.% titanium hydride (TiH_2_) powder was added, which produced hydrogen gas. Bubble formation resulted in the construction of a foam that had closed cavities, upon dispersing the particles in the melt at 700 °C for 3 min in the furnace to grow the core. The final phase of the foam was to cool the chamber to below the melting point of the alloy in order to freeze the melt before hydrogen getting out, or prior to the accumulation and cohesion of bubbles [27,29,31]. Next to this phase, the foam was removed from the mold for subsequent operations. As a result of the foaming process, large foamed blocks were obtained, which were subsequently cut into cubes to the desired dimensions (20 × 20 × 20 mm^3^).

**Figure 1:**
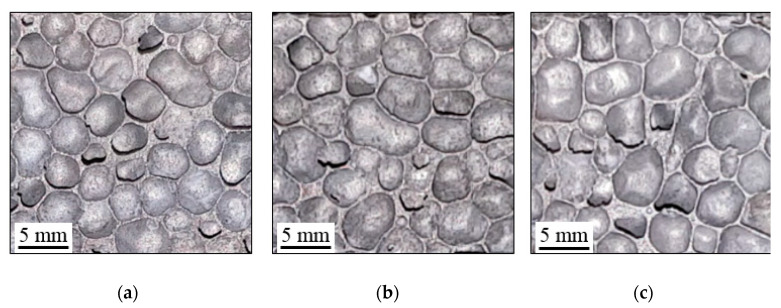
Foam structure according to loading direction: direction X (**a**), Y (**b**) and Z (**c**).

In order to obtain a defect-free cell structure, as shown in Figure 1, the AMF samples were prepared using wire electrical discharge machining. From Figure 1 it can be easily observed that the obtained foams highlight a closed-cell structure, having circular and elliptical shapes. From a simple visualization of the obtained images, on the three loading directions (LDs), it can be observed that the AMFs have remarkable differences in terms of microstructure, cell size and cell distribution. Therefore, it was expected that this aspect will influence the experimental results.

**Figure 2:**
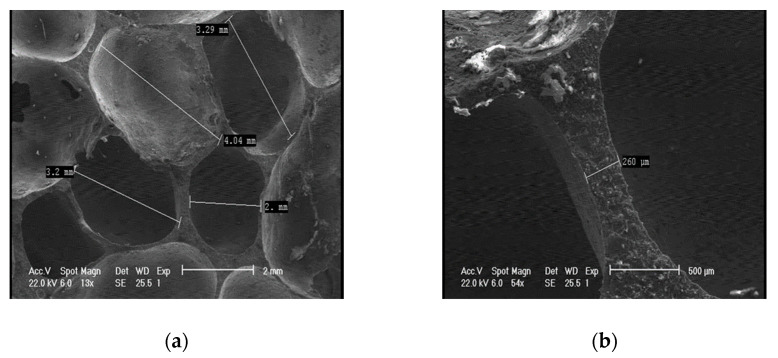
Morphology of aluminum metallic foams AMFs: shapes (**a**) and geometrical parameters (**a**,**b**) of the cells.

Figure 2 shows the SEM images of the AMF, with a detail on the shape and geometrical parameters (cell length and cell wall thickness) of the cells.

Due to some inherent irregularities such as micro-pores and other micro-defects, the average density of closed-cell AMFs was found to be around 0.52 g/cm^3^. Most pore sizes ranged from 2.5 mm to 4.5 mm, while wall thicknesses varied from 0.2 to 0.6 mm.

### 2.2. Experimental Tests Setup

The experimental program was performed by using a LBG TC100 universal test machine (LBG srl, Azzano San Paolo, Italy) equipped with a cooling compartment for low temperatures and a thermal chamber for high temperatures. The cooling compartment, together with a Liquid Nitrogen (LN) installation, were used for cryogenic temperature tests, while a thermal chamber was used for the high temperature tests. Tests at room temperature were performed on a universal compression device, being able to record also the deformation sequences of the AMF samples in real time, these images being used to define the collapse mechanisms.

**Figure 3:**
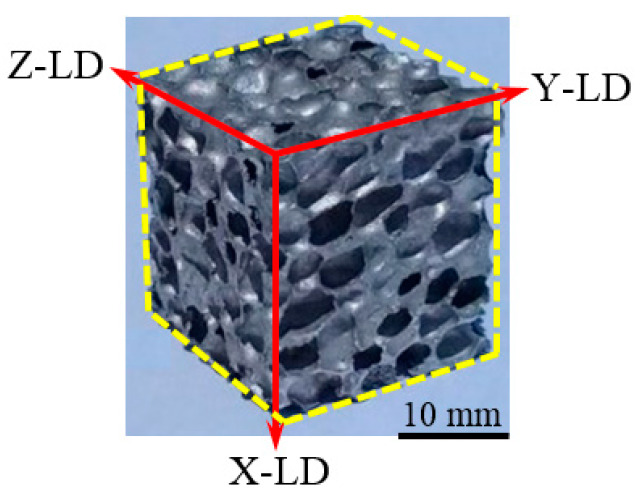
The foam sample together with loading directions (LDs).

Besides the different testing temperatures (−196, 25 and 250 °C), this paper also investigates foam anisotropy after the three loading directions (the X direction of the foam growth: out-of-plane loading; the Y and Z directions in the forming plan: in-plane loading). The anisotropy of the foam was investigated at all three mentioned temperatures. Figure 3 presents the obtained AMF cubic sample together with a detail of loading directions.

The experimental tests were performed according to the ISO 13314-2011 standard [32], using three samples for each condition. A constant speed of 10 mm/min was used during the compression tests for all investigated temperatures and loading directions. It is worth mentioning that, in order to obtain a homogeneous temperature distribution and reach an equilibrium in the foam structure, both the samples tested at the cryogenic temperature and those tested at the high temperature were kept in the appropriate device (cooling compartment for −196 °C and thermal chamber for 250 °C) for 10 min. The tests were performed inside the cooling/heating devices to avoid changing the temperature during the test (raising the temperature for cryogenic tests, and lowering the temperature for high temperature tests).

For an easier description of the results, the following notation convention was adopted in this paper: loading direction (X-LD, Y-LD or Z-LD)/testing temperature (CT, RT or HT), where CT stands for cryogenic temperature, RT for room temperature and HT for high temperature. As an example, X-LD/RT AMF corresponds to an aluminum metallic foam tested at room temperature for X loading direction.

## 3. Results and Discussions

**Figure 4:**
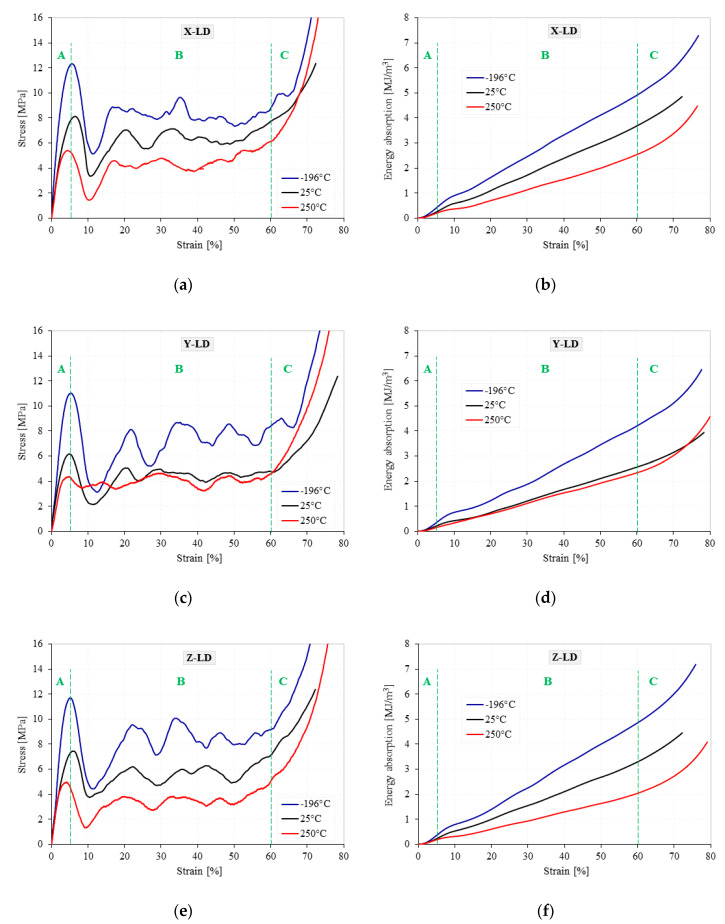
Stress–strain (**a**,**c**,**e**) and energy absorption–strain (**b**,**d**,**f**) curves of AMFs according to loading direction. Influence of testing temperature.

**Figure 5:**
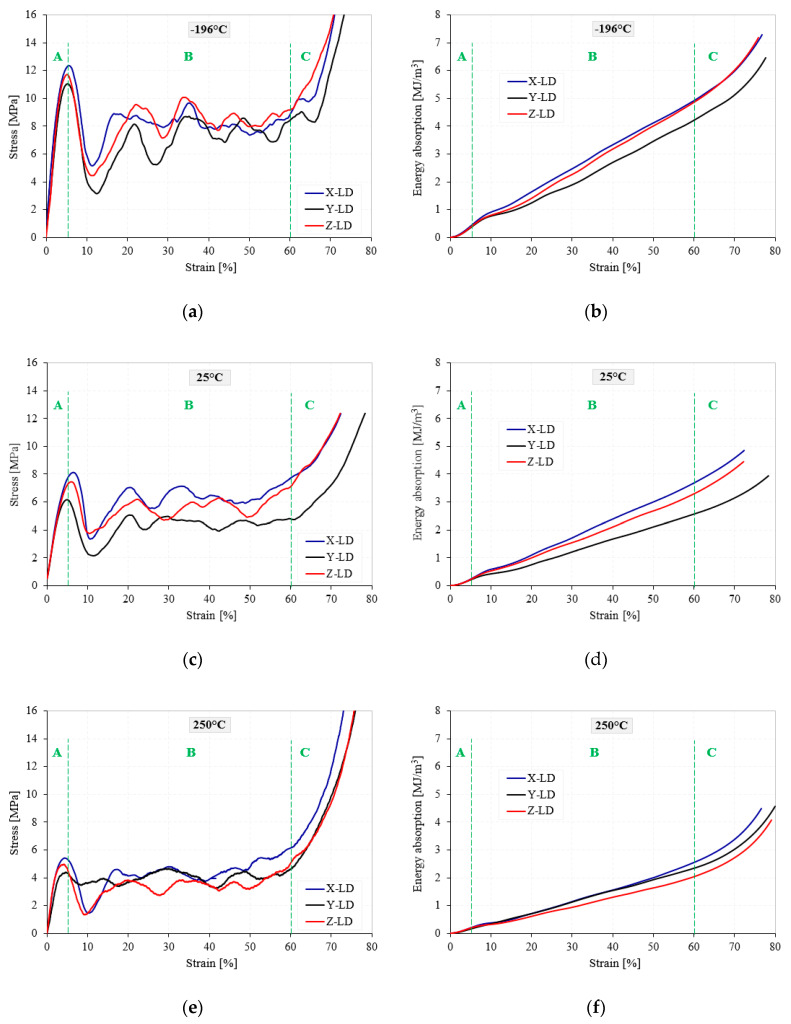
Stress–strain (**a**,**c**,**e**) and energy absorption–strain (**b**,**d**,**f**) curves of AMFs according to testing temperature. Influence of loading direction.

Following the experimental tests, the load-displacement data were recorded. Using the geometric parameters of the cubic AMF samples, the stress–strain curves and the corresponding energy absorption–strain curves were obtained and showed in Figure 4 and Figure 5 for all testing conditions.

The AMF samples were subjected to a compression test of about 80% strain, having a corresponding deformation of 16 mm. During the quasi-static compression tests, regardless of the loading direction and the testing temperature (TT), a typical behavior of closed-cell cellular materials, as usual, had been observed, highlighting three distinct zones with three different characteristics [33,34,35]. The curve starts with a *linear-elastic zone* (Zone A), where cell walls stretching and bending occurs and ends with the appearance of the first peak point, called yield stress. The second zone, *plateau zone* (Zone B), is characterized by the appearance of some stress fluctuations/oscillations in the early stages of compression. This phenomenon is probably due to repetitive cycles of cell-wall plastic collapse in the weak bands of the sample until the entire foam material reaches a second delimitation point, called onset strain of densification [36,37]. By increasing the TT, regardless of the LD, there is a significant reduction of the oscillations in the stress–strain curves. This considerable reduction in the number and amplitude of the oscillations from CT → HT is related to the softening of the AMF’s porous structure, due to the transition in foam behavior from brittle to ductile [24]. 

Finally, the curve ends with the *densification zone* (Zone C), where the stress increases considerably as a result of the forces exerted by the cell-walls and edges on each other, the foam material compacting like a solid material [38,39,40]. In addition, regardless of testing temperature and loading direction, all three zones have approximately the same order of magnitude in terms of deformation (0–5% strain for the linear-elastic zone, 5–60% strain for the plateau zone and 60–80% strain for the densification zone). On the contrary, it has been observed that both the TT and the LD significantly influence the stresses and energy absorption levels [41].

All stress and strain data were further processed following the ISO13314-2011 standard to determine the main mechanical properties [32]. Therefore, the following material properties were determined: first maximum compressive strength (or compressive yield stress) (*σ_y_*), strain corresponding to *σ_y_* (*ε_y_*), compressive stress at 20% and 40% macroscopic strain (*σ_20%_* and *σ_40%_*), plateau stress (*σ_pl_*), densification strain (*ε_d_*), and compressive strength (*σ_d_*) corresponding to *ε_d_*. Table 1 shows the main compressive properties of the foams according to loading direction and testing temperature.

The energy absorption (*W*) is represented by the area underneath the curves and was determined by integration of the stress–strain curve (Equation (1)), using variable integration limits [42,43,44]:

(1) $$W=\int_{0}^{50\%}\sigma d\varepsilon$$

The main values of *W* according to different testing temperatures (−196, 25 and 250 °C) and loading directions (X-LD, Y-LD and Z-LD) at some representative strains, using a 10% strain step, are presented in Table 2.

The *W* parameter is widely used in safety and impact engineering, especially in the automotive and building industries. From Figure 4 and Figure 5, it can be easily observed that a small amount of W is absorbed in the linear-elastic region (0–5% strain), while, due to the collapse of cell-walls, the largest amount of W is absorbed in the plateau region (5–60% strain) at an almost constant load. Therefore, zone B is very important for optimum choice of metallic foams used in the fields of energy absorption.

**Figure 6:**
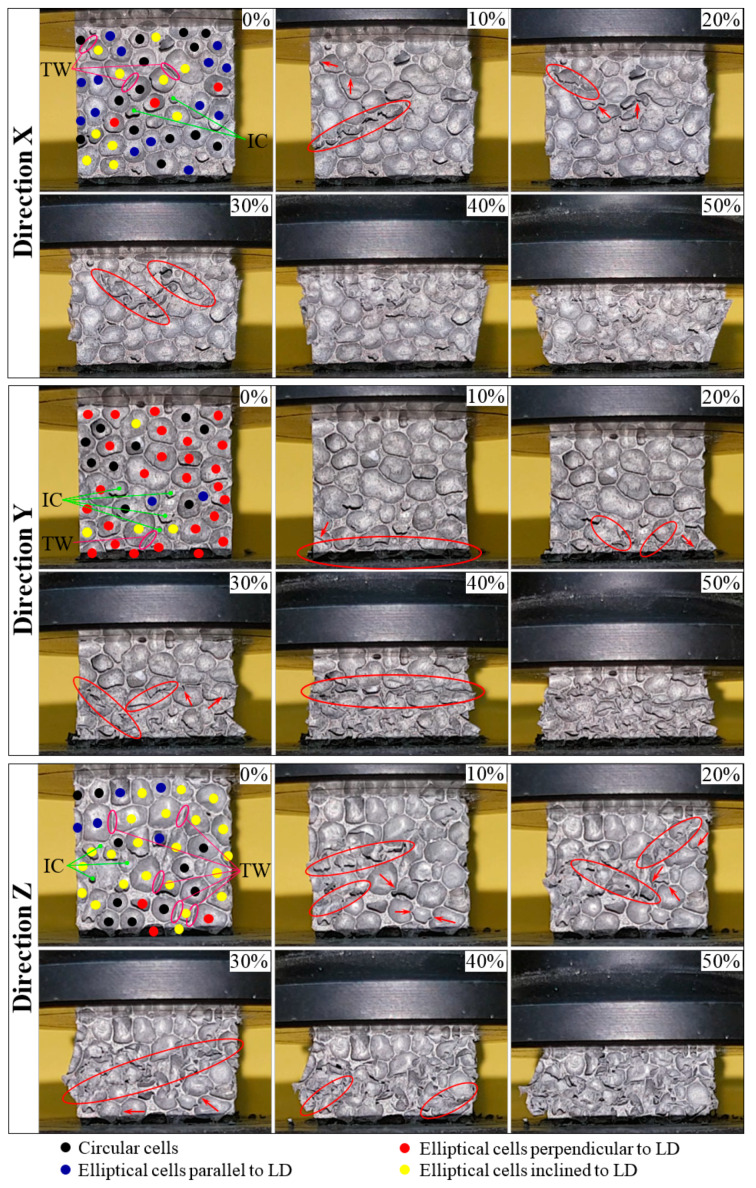
Deformation sequences of AMFs at room temperature according to loading direction.

Figure 6 shows the deformation sequences of AMF samples for the three loading directions (X-LD, Y-LD and Z-LD), at room temperature. It was not possible to present the collapse mechanisms at the other two testing temperatures (cryogenic and high temperatures) because the AMF samples were either completely submerged in liquid nitrogen (−196 °C) or completely closed inside the thermal chamber (250 °C). From Figure 6 (0% strain), it can be observed that, depending on the LD, the foam structure is predominantly governed by a certain type and orientation of cells.

**Figure 7:**
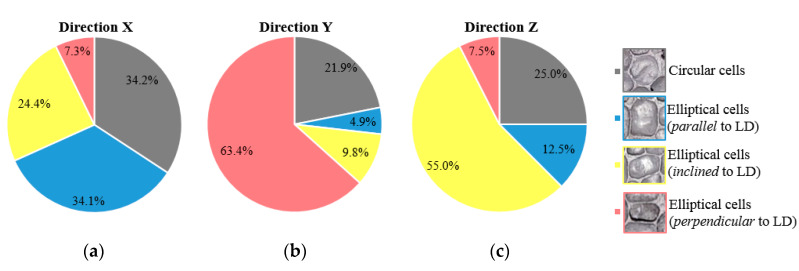
Percentage of cells’ distribution in foam structure for X-LD (**a**), Y-LD (**b**) and Z-LD (**c**).

For easier visualization of the cell type/orientation, Figure 7 presents the percentage of cells’ distribution in foam structure according to the AMF sample’s loading direction. During the deformation process of the AMF samples, it was observed that the circular cells (black dots from Figure 6) present the smallest role in the collapse mechanism process. In Figure 7, it can be seen that the circular cells are found in a percentage between about 22% and 34% in the microstructure of the foam. Contrariwise, the orientation of the elliptical cells significantly influence the deformation process. Therefore, the compressive foam strength for different loading directions is given predominantly by the orientation of the elliptical cells.

In addition, in the cubic samples, as can be seen in Figure 6 (at 0% strain), the intrinsic defects (such as intracellular cavities/micro-pores—IC, and thin cell walls—TW), that appear in the foam structure and their size, additionally contribute to the definition of collapse mechanisms. Whether the defective zones are perpendicular, parallel or inclined to the loading direction (according to foam formation), these micro-pores have a negative effect on the foam structure and, as the stress concentration, decrease the foam strength [45,46,47].

In the case of X-LD, the elliptic cells (about 34.1% of the total number of cells) are arranged in such a way that their large diameter are parallel to the direction of applying the compression load (blue dots from Figure 6) and can tolerate a higher stress (see Table 1). For the second loading direction (Y-LD), the large diameter of the elliptical cells (about 63.4% of the total number of cells) in the foam structure are perpendicular to the direction of the applied load (red dots from Figure 6). So, due to cell positioning, the foam would tolerate the lowest stress level compared to the other two loading directions (see Table 1). Finally, Z-LD is characterized by an arrangement of elliptical cells at a certain angle (the majority around 45°) to the direction of loading (yellow dots from Figure 6). Therefore, due to the inclination of the cells, the AMF samples will tolerate less stress than that of X-LD, as well as tolerating more stress than that of Y-LD (see Table 1). When AMF samples are subjected to a compression strain of 10%, defects such as intracellular cavities and thin cell-walls are the first places to start deformation, and they greatly reduce the mechanical properties. Further compression of these defects will make the deformation of the foam easier.

**Figure 8:**
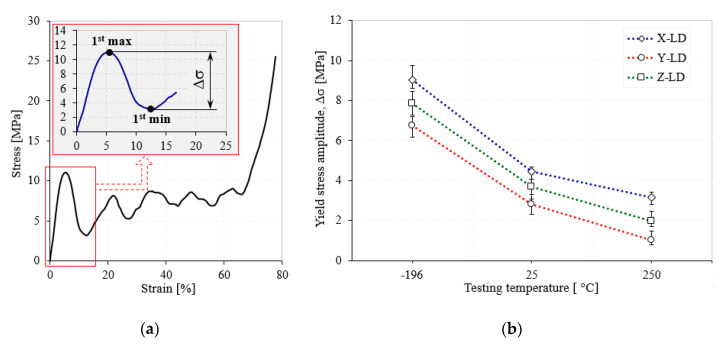
Detailed σ-ε curve (**a**) and Δσ-TT variation (**b**) of AMFs.

In the case of the cellular materials tested under compression loads, the yield stress is associated with their compression strength [48]. As can be seen in Figure 4 and Figure 5, the investigated metal foams show very large oscillations between the yield point and the onset strain of densification. Therefore, it is very important to associate these oscillations with the foam anisotropy for all investigated temperatures. The highest drop in the stress–strain curve is obtained immediately after the yield point, where the stress varies widely from a maximum (1st max) to a minimum (1st min) value. The difference between the 1st max and the 1st min stress is called the stress amplitude (Δσ) and is represented in detail in Figure 8a. To see more easily the influence of testing temperature and loading direction on the Δσ, Figure 8b shows the variation of the yield stress amplitude with the testing temperature, according to loading direction.

From Figure 8b it can be observed that, regardless of the loading direction, all the Δσ variations have the same pattern with the increase of testing temperature. The highest drop is obtained for the X-LD/CT (70.77%), while the smallest Δσ is obtained for the Y-LD/HT (23.23%). The major Δσ-drop is obtained from CT → RT (50.61% for X-LD and 52.93% for Z-LD), while the smallest from RT → HT (28.92% for X-LD and 46.49% for Z-LD). On the contrary, even if the Y-LD has lower values of the main properties (see Table 1 and Table 2), it appears that this direction is more stable during the deformation with respect to TT, thus having approximately equal values of the Δσ-drop from CT → RT (61.37%) and from RT → HT (63.70%). This pattern, from Figure 8b, confirms the results obtained in Figure 3 and Table 1 for yield strength values. Therefore, the microstructure of the foam significantly influences the collapse mechanisms and compressive strength of the AMF samples.

**Figure 9:**
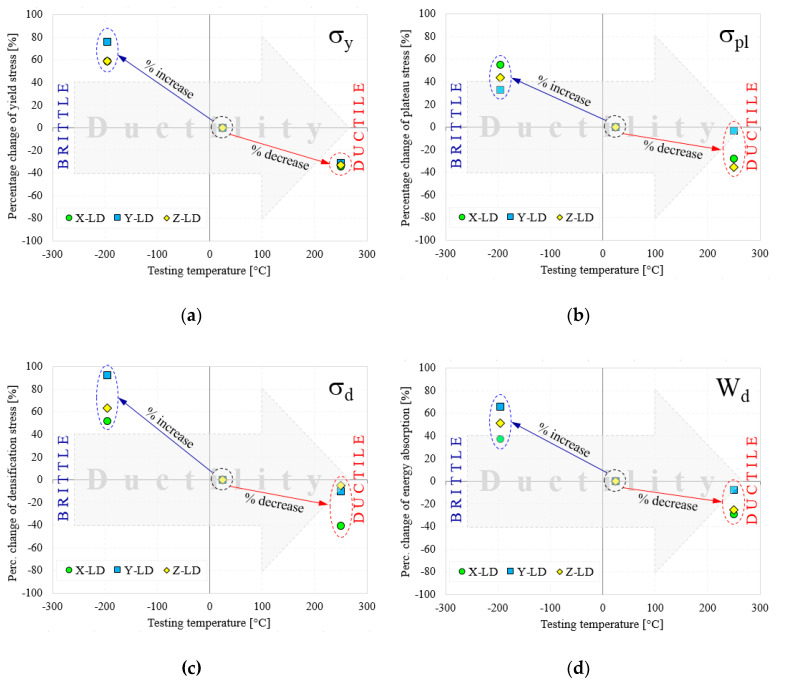
Percentage change of σ_y_ (**a**), σ_pl_ (**b**), σ_d_ (**c**) and W_d_ (**d**) according to testing temperature and loading direction.

According to testing temperature and loading direction, Figure 9 compares the relative percentage change (RPC) of the normalized yield stress, plateau stress, densification stress and the energy absorption for AMF samples. The strength properties and energy absorption performances were normalized according to the experimental results obtained at room temperature [49]. Therefore, according to testing temperature, the RPC is represented by a relative percentage increase (RPI) in the CT tests and a relative percentage decrease (RPD) in the HT tests.

With the decrease of testing temperature, from RT → CT, a significant increase of the properties is obtained. Interestingly, at −196 °C, even if the Y-LD has much smaller properties than the X-LD, the RPI of the X-LD/CT sample is significantly below the RPI of the Y-LD/CT sample (see Figure 9a,c,d). The only exception is made by the normalized plateau stress, which has higher RPI on X-LD, compared to Y-LD (see Figure 9b). In all cases, RPI after Z-LD is found between the other two RPIs. It seems that the inclined distribution of the elliptical cells in the foam microstructure leads to a stable deformation of the Y-LD AMF samples and also to the constant maintenance of the RPI during the compression tests performed at CT. On the other hand, with increasing test temperature, from RT → HT, a decrease in properties was observed. The normalized yield stress has approximately the same RPD value for all loading directions (Figure 9a). As in the case of RPI, the smallest RPD was obtained for Y-LD, while similar values were obtained for X-LD and Z-LD (Figure 9b,d). Regarding the normalized densification strain, at the 250 °C, the RPD of the Y-LD samples is only slightly below the X-LD sample. It seems that at HT, the inclined elliptical cells of the AMF are deformed much more irregularly than in the CT case, leading to a rather scattered distribution of RPD compared to RPI.

Therefore, it is very important to select the type of used AMFs (more precisely the selected loading direction), depending on the operating temperatures (CT, RT or HT) of the assembly to which it belongs. If the assembly works under normal temperature conditions (RT), it is recommended to use the X-LD AMFs, as it has much better strength properties and energy absorption performances than the other two (Y-LD and Z-LD). Otherwise, if the assembly works under extreme temperatures (CT or HT), it is recommended to use Y-LD AMFs, as it has the highest RPI, and the smallest RPD, compared to X-LD and Z-LD.

## 4. Conclusions

This paper investigates the anisotropic compressive behavior of aluminum metallic foams AMFs under extreme temperature conditions (−196 °C → 250 °C). For this purpose, cubic AMF samples were manufactured and tested according to different loading directions (LDs) and different testing temperatures (TTs). As a result of these investigations, the following aspects can be concluded:

▪ Following the casting process, the AMF samples show a structural (geometrical) anisotropy with circular and elliptical cells of different sizes and orientations (Figure 1, Figure 6 and Figure 7). In addition, different intrinsic defects (intracellular cavities, micro-pores and thin cell walls) are found in the microstructure of the AMFs (Figure 6). These geometrical characteristics are essential for quantitatively rigorous predictions of main mechanical properties.
▪ Regardless of the loading direction, the TT significantly influences the compression behavior of the AMFs. Due to the softening of the matrix, the mechanical properties significantly decrease with the increase of the testing temperature, and the collapse mechanism changes from a brittle one (−196 °C) to a ductile one (250 °C).
▪ Regardless of the testing temperature, the mechanical property anisotropy is governed by the AMF’s microstructure. It was found that the elliptical cells, arranged in such a way that their large diameter are parallel to the direction of applying the compression load (abbreviated X-LD), can tolerate a higher stress compared to Y-LD and Z-LD (Table 1, Figure 4 and Figure 5).
▪ Due to the brittle–ductile transition with the increase of the TT a decrease in stress amplitude (Δσ) is obtained. The highest Δσ is obtained for the X-LD, while the smallest one for the Y-LD.
▪ The relative percentage change of normalized compressive properties significantly differs depending on the loading direction and testing temperature. For temperatures higher than RT, a percentage increase is obtained, while for temperatures lower than RT, a percentage decrease is obtained. The highest percentage increase (at −196 °C), and the smallest percentage reduction (at 250 °C) is obtained for Y-LD.

## Figures and Tables

**Table 1:** Compressive properties of AMFs according to loading direction and testing temperature.

Loading Direction	TT [°C]	$\sigma_{y}$ [MPa]	$\varepsilon_{y}$ [%]	$\sigma_{20\%}$ [MPa]	$\sigma_{40\%}$ [MPa]	$\sigma_{pl}$ [MPa]	$\sigma_{d}$ [MPa]	$\varepsilon_{d}$ [%]
X-LD	−196	12.20	4.19	4.19	7.67	9.26	9.05	58.23
	25	7.68	6.18	6.18	6.26	5.98	5.95	60.32
	250	5.07	3.21	3.21	3.88	4.32	3.55	65.42
Y-LD	−196	10.69	4.36	7.40	6.47	5.81	8.41	56.50
	25	6.08	5.09	4.88	4.33	4.37	4.37	58.30
	250	4.18	3.61	3.63	3.35	4.23	3.92	62.85
Z-LD	−196	11.47	4.33	8.26	8.46	8.27	8.09	57.34
	25	7.22	5.87	5.73	5.75	5.76	4.96	59.09
	250	4.82	3.63	4.40	3.51	3.72	4.71	61.77

**Table 2:** Energy absorption values of AMFs according to loading direction and testing temperature.

Loading Direction	TT [°C]	Energy Absorption at Different Strains [MJ/m^3^]						
		10%	20%	30%	40%	50%	60%	70%
X-LD	−196	0.87	1.46	2.33	3.14	3.92	4.92	5.97
	25	0.52	1.01	1.63	2.31	2.93	3.59	4.45
	250	0.36	0.70	1.13	1.55	1.99	2.54	3.37
Y-LD	−196	0.76	1.23	1.90	2.69	3.45	4.20	5.11
	25	0.40	0.71	1.17	1.64	2.37	2.53	3.09
	250	0.33	0.65	1.08	1.49	1.92	2.34	3.02
Z-LD	−196	0.78	1.34	2.18	3.01	3.90	4.89	5.87
	25	0.49	0.94	1.50	2.05	2.63	3.23	4.10
	250	0.35	0.68	1.10	1.51	1.95	2.41	3.15
